# Gamma band LFP in mouse barrel cortex is coupled to respiratory rhythm

**DOI:** 10.1186/1471-2202-13-S1-P123

**Published:** 2012-07-16

**Authors:** Junji Ito, Snigdha Roy, Ying Cao, Sonja Grün, Detlef Heck

**Affiliations:** 1 Institute of Neuroscience and Medicine (INM-6), Forschungszentrum Jülich, Jülich, Germany; 2 Department of Anatomy and Neurobiology, University of Tennessee Health Science Center, Memphis, TN, USA; 3 Theoretical Systems Neurobiology, RWTH Aachen University, Aachen, Germany

## 

**Figure 1:**
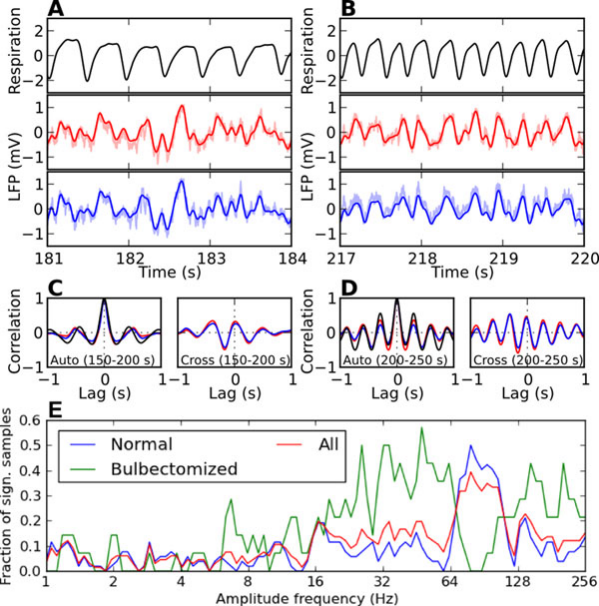
**A.** Respiration (top) and LFP (bottom two) traces during normal breathing. The respiration signal was recorded with a thermistor and its unit is arbitrary. LFP recording sites were 610 um apart. Solid curves are the signals after band-pass filtering in 0.5-10 Hz. Raw LFP signals are plotted with shaded colors. **B.** Same as **A**, but during accelerated breathing after hypoxia. **C.** Auto-correlation of the respiration and the LFP signals (left) and cross-correlation between the respiration signal and each of the LFP signals (right) during normal breathing. **D.** Same as **C**, but during accelerated breathing. **E.** Summary of phase-amplitude coupling analysis on the LFP data. The strength of the coupling between the phase of the respiratory frequency and the amplitude of a range of frequencies between 1 and 256 Hz was assessed in terms of the mean vector length. Analysis was performed on 52 and 14 LFP segments (duration: 50 sec) from normal and bulbectomized mice, respectively. The fraction of significant samples is plotted as a function of amplitude frequency.

The neocortex of mammalian brains shows prominent oscillatory activity in the local field potential (LFP) and EEG signal within a broad range of frequencies from slow (1-8 Hz) delta/theta band to gamma band (40-100 Hz) and higher frequencies [1]. Oscillations in different frequency bands seem to be linked. There is increasing evidence that amplitude modulations in the gamma frequency band are phase-locked to the delta/theta rhythm [2]. Several studies have linked theta-gamma phase-amplitude coupling to cognitive processes [3]. Here we report that in awake mice gamma band LFP power in the barrel cortex is phase-locked to the concurrent theta band LFP oscillation and that this theta band oscillation is strongly correlated to the respiratory rhythm. When the animals were briefly exposed to hypoxic air, the resulting frequency increases in the respiratory rhythm were paralleled in the delta/theta band LFP oscillations (Fig.1A-D). LFP oscillations in sub-bands of the broad (40 – 100 Hz) gamma frequency band were amplitude-modulated in phase with the breathing frequency (Fig. 1E). After removal of the olfactory bulb the frequency profile of the phase-amplitude coupling was significantly changed. Particularly the respiration-locked amplitude modulation in the high gamma band (64-128 Hz), which was prominent in healthy control mice, shifted to lower frequencies in bulbectomized mice. Our findings imply that in mice respiratory activity directly modulates delta/theta band LFP oscillations through respiration-locked olfactory bulb activity and indirectly, through phase-amplitude coupling, gamma band power.
